# Current and prospective control strategies of influenza A virus in swine

**DOI:** 10.1186/s40813-021-00196-0

**Published:** 2021-02-28

**Authors:** Hamish A. Salvesen, C. Bruce A. Whitelaw

**Affiliations:** The Roslin Institute and Royal (Dick) School of Veterinary Studies, University of Edinburgh, Easter Bush, Edinburgh, UK

**Keywords:** Swine, Influenza, Pandemic, Disease control

## Abstract

**Background:**

Influenza A Viruses (IAV) are endemic pathogens of significant concern in humans and multiple keystone livestock species. Widespread morbidity in swine herds negatively impacts animal welfare standards and economic performance whilst human IAV pandemics have emerged from pigs on multiple occasions. To combat the rising prevalence of swine IAV there must be effective control strategies available.

**Main body:**

The most basic form of IAV control on swine farms is through good animal husbandry practices and high animal welfare standards. To control inter-herd transmission, biosecurity considerations such as quarantining of pigs and implementing robust health and safety systems for workers help to reduce the likelihood of swine IAV becoming endemic. Closely complementing the physical on-farm practices are IAV surveillance programs. Epidemiological data is critical in understanding regional distribution and variation to assist in determining an appropriate response to outbreaks and understanding the nature of historical swine IAV epidemics and zoonoses.

Medical intervention in pigs is restricted to vaccination, a measure fraught with the intrinsic difficulties of mounting an immune response against a highly mutable virus. It is the best available tool for controlling IAV in swine but is far from being a perfect solution due to its unreliable efficacy and association with an enhanced respiratory disease. Because IAV generally has low mortality rates there is a reticence in the uptake of vaccination.

Novel genetic technologies could be a complementary strategy for IAV control in pigs that confers broad-acting resistance. Transgenic pigs with IAV resistance are useful as models, however the complexity of these reaching the consumer market limits them to research models. More promising are gene-editing approaches to prevent viral exploitation of host proteins and modern vaccine technologies that surpass those currently available.

**Conclusion:**

Using the suite of IAV control measures that are available for pigs effectively we can improve the economic productivity of pig farming whilst improving on-farm animal welfare standards and avoid facing the extensive social and financial costs of a pandemic. Fighting ‘Flu in pigs will help mitigate the very real threat of a human pandemic emerging, increase security of the global food system and lead to healthier pigs.

## Background

**Fig. 1:**
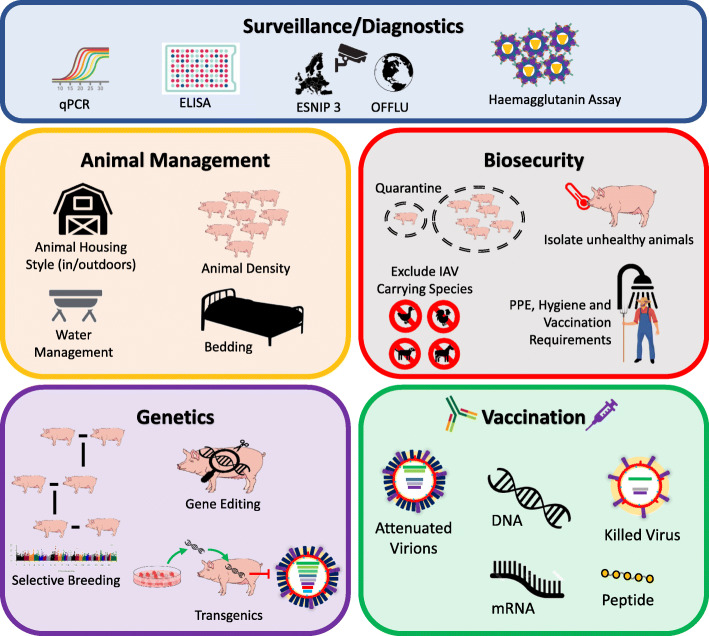
The current and prospective strategies available for influenza A virus control

Influenza viruses are significant pathogens of humans, livestock and a multitude of wild species. They have a diverse and complex ecology stemming from their ability to cross species barriers. Comprising four genera within the *Orthomyxoviridae* family, Influenza A Virus (IAV), Influenza B Virus (IBV), Influenza C Virus (ICV) and Influenza D Virus (IDV) are enveloped virions with segmented negative sense RNA genomes (Fig. 1a). Seasonal epidemics of IAV and IBV occur in humans whilst only IAV has been attributed to cause epidemics in swine [1]. IBV [2], an ICV-related pathogen [3, 4] and IDV [5] have been reported and associated with mild morbidity in domestic pigs [4, 6]. Herein, the focus will be on IAV due to its more significant historical impacts and greater potential for emergence as a swine or human pandemic.

As in humans, swine infections with annual epidemic strains are generally associated with high morbidity and low mortality [7]. Swine IAV (swIAV) causes respiratory illness that is clinically characterised by fever, loss of appetite and coughing. These symptoms cause weight loss, which significantly affects the productivity of growing pigs and the reproductive performance of breeding sows [7, 8]. An epidemiological meta-analysis determined the global pig and herd-level seroprevalence as 49.9 and 72.8%, respectively. A significant proportion of the over 1.4 billion pigs in 2018 have therefore been affected by swIAV [9, 10]. Because swIAV is usually observed as a component of Porcine Respiratory Disease Complex (PRDC), the reduced productivity and animal welfare from swIAV infection are compounded by pathogenic co-infections [11, 12]. The presence of viral infections are a significant influence on the profitability of a farm [13].

In the USA, costs per finishing pig were estimated to be $10 when PRDC co-infections are considered, representing a significant loss of income [14]. The major economic concerns for hog farmers from swIAV stem from the reduced productivity that entails a longer time to slaughter and the lower number of average piglets per sows in IAV endemic herds [15]. A German study identified that 80% of farms with clinical presentation of swIAV had reduced reproductive performance prior to implementation of a vaccination program and found higher abortion and preweaning mortality rates that result in the average of piglets weaned per sow annually being reduced by more than one, a factor that will seriously impact the economic performance of affected farms [8].

The 2009 pandemic H1N1 (pH1N1) outbreak, eponymously named “Swine ‘Flu”, provides a warning of the potential damage to the pork industry of indirect costs that are incurred from swIAV zoonotic transmission. Furthermore to the direct costs, public misconceptions about the safety of eating pork and concerns of sustained swine to human transmission caused losses to the US pork industry estimated to be over $1 billion USD [16] and a reduction in Mexican GDP of over $3.2 billion USD [17] was incurred from trade embargoes and consumer abstention. These examples demonstrate the fragility of consumer pork demand in the instance of a swine origin pandemic outbreak.

The IAV genome consists of 8 RNA segments that encode for at least 12 proteins (HA, NA, PB1, PB1-F1, PB2, PA, PA-X, NP, M1, M2, NS1, NEP) [18, 19]. Nomenclature for IAV derives from antigenic subtypes determined by the major surface glycoproteins haemagglutanin (HA) and neuraminidase (NA). The genomic architecture of IAVs is essential to understanding its history and continued evolution in intraspecies and zoonotic scenarios. In the instance of two distinct IAVs co-infecting a single host cell, genomic segments can become reassorted, leading to antigenically novel viruses budding, with progeny virions having a different genomic composition to the ancestral infectious particles [20, 21]. Termed antigenic shift, this phenomenon supports rapid generation of novel IAV subtypes, that in turn promotes the circumvention of the host immune response by evading recognition [22].

Genome reassortment alone does not drive IAV emergence, with the error-prone viral RNA-dependent RNA polymerase (RdRp) contributing through the introduction of mutations during RNA replication [23]. In a constant evolutionary arms race between IAV and their hosts, the adaptive pressure exerted by host immune systems upon the virus is the response that stimulates IAV phenotypic diversity and ultimately drives their evolution. Lacking a proof-reading function, diversity in the viral genome gradually accumulates, providing a source of variation for the forces of natural selection to be imposed on, in a process known as antigenic drift (Fig. 1b) [24]. The viruses that can transmit between hosts and replicate within a host most efficiently then become pervasive by superior propagation. Understanding the genetic evolution of IAV is critical to disrupting the IAV ecosystem and determining the approach of controlling both intraspecies and zoonotic transmission.

Livestock species, primarily pigs and chickens, and the persistent IAV natural reservoir of wild waterfowl play a central role in the ecology of IAV. The close phylogenetic relationship of chickens and waterfowl means that there are fewer biological hurdles for avian IAV zoonotic events. Opportunity for cross species transmission arise in scenarios such as wild birds mixing with free range chickens, with the regular global movements of birds on migratory travel allowing widespread dispersal [25]. However, avian adapted IAV subtypes face greater barriers for transmission into mammalian species due to biological differences between hosts. For example, a well characterised barrier to avian IAV transmission to humans is the reduced capacity of avian adapted strains to bind to and enter human epithelial cells effectively [26]. The IAV glycoprotein HA binds specifically with sialic acid (SA) to trigger receptor mediated endocytosis entry into host cells. The prevalent SA isoforms in humans (α2–6) and chickens (α2–3) are well characterised determinants of species specificity, with specific amino acid changes in HA of avian derived IAVs being associated with an increased potential for the emergence of a human pandemic [27, 28].

The respiratory tract of pigs has both α2–6 and α2–3 on the surface of epithelial cells, creating the opportunity for both avian adapted and mammalian adapted IAVs to enter the same cellular environment [29, 30]. In the occurrence that multiple IAV subtypes concurrently infect a single host cell there is the potential for novel viral emergence by antigenic shift, which has led to pigs being coined as “mixing vessels” (Fig. 1c) [31, 32]. Spill overs of avian origin IAV into porcine hosts occurs frequently, however transmission between pigs is then limited without further mammalian adaptations [33]. This is because following successful entry of IAV into the host cell, other key steps including transport of viral RNA into the nucleus [34, 35] and replication of the viral genome within the nucleus [36–38] require adaptations to the host environment. Because of IAVs error-prone RNA replication, beneficial genetic changes can be rapidly acquired in the new host if completion of the full viral cycle is possible. The infectious ability of IAV is associated with signature amino acid substitutions that are acquired following zoonotic transmission [39].

For 80 years there was a single known strain of swIAV in North America, the 1918 H1N1 strain (cH1N1). Our inability to control swIAV in a globalised world has provided the basis for further evolution and divergence into distinct clades within each subtype. H3N2 emerged in 1998 as a result of triple genome reassortment with HA, NA and PB1 from human seasonal influenza, PB2 & PA from and avian IAV and NP, M1, M2, NS1 & NEP from cH1N1. The acquisition of this triple reassortment gene cassette (TRIG) has driven further emergence of multiple strains (H1N1, H1N2 & H3N2) that are now globally endemic in pigs [9, 40]. H3N1 has emerged most recently in Europe, Asia and the United States but has not yet been associated with large outbreaks [41–43].

The outbreak of pH1N1 in 2009 brought the role of pigs in IAV ecology to the fore. Despite their role not being clearly understood, it was quickly appreciated that the human-swine interface played a major role not only in regard to the original emergence, but through continued bidirectional swine-human transmissions [44]. Distinct antigenic derivations emerged in North and South America, Asia and Europe as a result of divergent evolution following establishment in local swine populations [21, 40]. Regional divergence and reassortment events of pH1N1 exemplifies how genetic drift and selective sweeps affect the evolution of IAV [45]. Recent examples of further swine-human transmission include an H3N2 strain containing the pH1N1 matrix protein (M1) that has largely been detected in attendees of agricultural fairs in the USA, and seropositivity in Chinese swine workers against a novel strain reassorted from avian-like H1N1 and pH1N1 identified [46, 47]. Given that these examples are from regions with existing swIAV surveillance infrastructure, where bidirectional human-swine IAV transmission has not been identified it should be considered whether it is genuinely a case of it not occurring or whether it has just not yet been detected.

With pig farming becoming more industrialised to meet consumer demands for pork, the higher density of pigs is likely to increase swIAV prevalence if left uncontrolled [9]. Close and regular interactions between swIAV endemic pigs and humans creates an environment that could lead to the emergence of novel strains through bi-directional transmission and subsequent reassortment events [48–50]. Furthermore, the limited but present international trade of pigs and global movement of people exacerbates this potential for co-infection with multiple distinct IAV strains [51, 52].

**Fig. 2:**
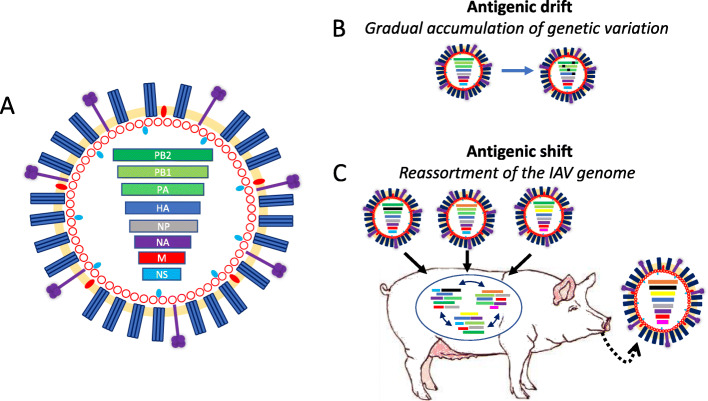
Schematic diagrams of influenza A virus and its evolution. **a**) The influenza A virion is an enveloped virus containing 8 negative sense singlestranded RNA segments. **b**) Genetic variation accumulates in the IAV genome during RNA replication. **c**) Novel IAVs can arise through reassortment of the 8 genomic segments during co-infection of a single host

In this review we consider current and prospective control strategies that aim to reduce the prevalence of swIAV across all pig farming systems in the face of these increasing threats (Fig. 2). Each farming system will have differing practicalities and cost/benefits prospects for each method, and therefore it is only suggested that farms implement the maximum that is possible in an effective way within their system should be done to control swIAV.

### Current control measures

To control the spread of swIAV, the number of onwards infections must be reduced to be below 1 per infected animal. The number of onwards infections per animal in an entirely susceptible population is known as an R0 value. If it is below 1 the pathogen will subside in a population. Control strategies can be reactive, which aim to clear a virus infection after clinical presentation or detection, or proactive, which aims to prevent a pathogen becoming established. With swIAV having an R0 value of 10.66 it will rapidly spread in if no control programs are implemented [53]. Effective control is vindicated through improved future economic performance on swIAV free farms as a result of better productivity efficiency and reduced veterinary costs.

From a human health perspective, the reduction in swIAV prevalence will lower the potential for the emergence of a pandemic, which have historically had huge societal and economic impacts. Controlling IAV in swine should be approached with ‘One Health’ considerations due to its significant role in humans, livestock and the environment (wild species). As a quintessential zoonotic pathogen, successful control in one species will have knock-on effects throughout the swIAV ecological web. All control measures discussed here refer to their application in farmed pigs, as although wild pigs are infected [54–56] they cannot be managed in the same manner and the low prevalence and minimal interactions with humans or pig farms means they present a low risk. Reduced swIAV prevalence may also benefit by less erroneous use of antibiotics due, which has unfortunately been rising alongside industrial farming practices [57].

### Animal management

The most basic swIAV control strategy is through evidence-based animal husbandry methods that will concomitantly benefit animal welfare standards. Animal management control measures are most effective when applied pre-emptively to prevent swIAV establishment rather than retrospectively to clear an endemic outbreak [58, 59]. With high levels of subclinical presentation and variable results from medical interventions, once an outbreak has begun it can be difficult to intervene.

In favourable conditions, such as in cold water or with cold temperatures on a hard surface, IAV can remain infectious outside a host for beyond a year [60, 61]. The increased persistence in water is particularly relevant as Mastin et al. (2011) [58] observed reduced odds ratios for swIAV in pigs where there were less than 18 finishers for each water access. The main route of transmission postulated is via droplets [62–64] during physical contact with another pig or from contaminated surfaces, so it is expected that increasing the incidences of nasal contact and the sharing of mucus will assist the perpetuation of swIAV. As follows, the size and density of pig herds is a risk factor for increased prevalence [65–67]. Furthermore, overcrowding creates stressful conditions that can lead to a depression of the immune response that increases the susceptibility and severity of swIAV across a herd [68]. The type of farm and housing system also plays a role in swIAV epidemiology. Measures such as using straw for bedding as opposed to having a slat floor system have been associated with a lower seroprevalence [58, 65], whilst indoor housing [58] and open partitions between pens in barns [69] have been associated with an increase. With indoor housing, slat floor systems and high-density herds increasingly common, these factors may contribute to the increasing swIAV prevalence observed on pig farms.

Effective animal management includes collecting data on husbandry and animal movements for use in supporting effective trace and isolate protocols as required. If an outbreak occurs, movement restrictions can then be applied quickly, but only if appropriate information is available to make an informed decision. Regarding movements, fallow periods for pens are recommended but are not realistic to apply in economically optimised systems. More feasible is sanitation of pens between groups with disinfectants that inactivate viruses to ensure transmission does not happen between groups on arrival in a new pen. Disinfecting pens further benefits by killing other pathogens of PRDC [70]. In a worst-case scenario, the culling of entire herds and having a fallow period to clean farms is a viable method of animal management to eradicate disease, however the repercussions of eradicating pigs could not be more detrimental financially or emotionally to farmers. These drastic measures were taken in response to pH1N1 in only Norway and Egypt, primarily as preventative measures for swine-human transmission, however it was not considered widely effective as human-human transmission was significantly the main source of infection [71]. In 2009 the World Health Organisation (WHO) announced that trade restrictions on pigs or pork products were unnecessary, but reflecting this decision with our current knowledge that bidirectional transfer can occur, the continued movement of pigs which disseminated the virus between pigs [44, 72] may also have posed a limited but present threat to humans who had close contact with swine farms.

### Biosecurity

As well as on-site management of animals, swIAV control must account for how multi-site pig systems require regular movement of pigs between stratified breeding farms through to finishing farms. Intuitively, movement of an infected animal into a naïve herd is a primary source of infection, and so once pathogen free status is attained the aim is to prevent reimportation. To reduce the likelihood of swIAV establishing in a naïve population from new arrivals, quarantining before mixing with the original herds is recommended since the presentation of clinical symptoms occurs subsequently to the peak of time of viral shedding [73]. Given that many animals are asymptomatic carriers that present subclinical infections [7, 74, 75], a lack of visible symptoms should not be considered definitive confirmation of no infection and excuse of quarantine before clearance for herd integration.

All-in-all-out systems would reduce the risk of new arrivals becoming infected; however, the logistical complexity and dynamism of pig breeding does not lend itself to this being practical. If the breeding farms pathogen status is known, receiving farms can appropriately have either confidence or mitigation strategies in place. Given the time and cost of using the available diagnostic tests, and the potential for subsequent infection between testing and movement, quarantine is the most effective blanket measurement to take for new arrivals.

Observations that higher replacement rates are associated with higher seroprevalences of swIAV outlines the potential risk of farming with high replacement rates that are inherently necessary in multi-site farming systems [66, 69]. However, the impact of replacement is not well defined, as data from swine belt states in the USA observed no reduction of endemic swIAV prevalence associated with the closure of breed to weaning herds, suggesting further work is needed to understand whether inter-herd transmission or within herd transmission is the main driver infectious cycles [59].

The inherent movement requirements of multi-site production systems mean the entire system is more efficient with higher localised farm densities to reduce transport costs and permit hubs of abattoirs and feed production. Although beneficial for ease of coordination and economic efficiency, proximity to other hog farms and density of pig farms has been positively associated with increased swIAV seroprevalence [66, 67]. An unseen risk of farm density is present in barn exhaust air that has purportedly been detected to still contain swIAV over 1 mile away [40]. With multi-site pig production systems expected to become increasingly popular, these conditions that are more permissive to swIAV transmission will lead to a higher prevalence [9]. Beyond regional boundaries, international transport of live pigs encourages the global dissemination of swIAV [72, 76, 77], with North American and European swIAV lineages now circulating in China and Africa [43, 44].

Biosecurity control strategies should not be limited in their focus to pigs. Workers on swine farms are regularly handling pigs and there is therefore potential for transmission of IAV between pigs and humans [44, 50, 78]. If an employee or pig is infected with an endemic strain to their species and then a zoonotically transmitted strain, genomic reassortment can occur. Restricting entry onto pig farms of only essential people (*e.g* vets, employees, suppliers), ensuring all employees and their families are vaccinated, and implementing good hygiene across all farm practices will help reduce the risk of bidirectional transmission and the emergence of a novel strain by zoonoses [79]. Good hygiene practices include wearing Personal Protective Equipment (PPE) and strictly enforcing ill employees not to attend farms. A lack of biosecurity measures can be seen to have an effect as agricultural fairs in the USA are an identified hotspot for swine-human transmission [9, 46, 80]. The close contact required for showing pigs, minimal attempts at mitigation compared to the farm setting and the mixing of pigs from distinct origins creates the ideal opportunity for IAV strain admixture and bidirectional transfer.

Biosecurity protocols should also consider the threat of IAV infections from beyond humans and pigs. There have been instances of what were thought to be swIAV free herds in well ventilated and secure barns detecting avian influenza strains [81–83]. It was noted following detection of avian IAV strains in these herds that they use surface water from nearby ponds to clean out the pens between replacing stock. As the surface water was used by ducks and other waterfowl species that are known to excrete IAV in their faecal matter, it is plausible the water acted as the vector for IAV transmission [60, 61]. The biosecurity hazard of pooling water near-by, especially when used as a resource for cleaning pens exemplifies that anything entering a pig farm could be considered a risk for swIAV introduction.

For small scale holders the practical biosecurity measures differ from indoor systems. Although indoor housing systems have been observed to assist in harbouring endemic swIAV [9], on outdoor farms there is a scenario of not being able to exclude other IAV susceptible animals such as birds, cats and mustelids which creates a cauldron for IAV mixing from multiple hosts. However, for either farming system, knowing where breeders come from, quarantining new arrivals, receiving information of the previous owners swIAV management protocols and maintaining good hygiene practice where possible will contribute to reducing the prospect of swIAV becoming endemic.

### Surveillance

Because of the low mortality, high morbidity and generally self-limiting nature of swIAV infections, it is not considered as a notifiable disease by the World Organisation for Animal Health (OIE) or the United States Department of Agriculture (USDA) [84, 85]. However, surveillance data optionally collated does help to understand the epidemiology and evolution of swIAV and can be applied to inform policy decisions. Because of its diversity and intrinsic mutability, knowledge of the prevalent genetic and antigenic landscape enhances the effectiveness of control measures in place. The more we understand about swIAV, the more targeted control measures can be. Up to date information on swIAV’s distribution and prevalence also plays an important role in ensuring that current vaccines remain relevant to the prevailing endemic strains [86].

Although knowledge acquired from surveillance does not directly control swIAV, the timely sharing of virology and epidemiology data to identify infection hotspots and transmission events within and between herds/species, allows us to gain insight into where novel strains are likely to emerge and respond to prevent further transmission and establishment in a population [80, 86]. Because of this the USDA established a national swIAV surveillance program after 2009 [87] and Europe followed suit with the European Surveillance Network for Influenza in Pigs (ESNIP) [88]. Further supporting surveillance is the establishment of OFFLU (www.offlu.net), a collaborative effort of experts on animal influenza aiming to promote animal influenza research and data sharing that is supported by the OIE and FAO [89]. Given that the dynamics of swIAV infections have been reported to be both cyclical as in humans [59, 90], and persistent throughout the year [91], we should be aware that swIAV epidemiology is not necessarily directly translatable from human research.

Despite the overt swine health and swIAV control benefits derived from mass surveillance, efforts are sometimes impeded by concerns that public knowledge of the distribution or identification as a swIAV hotspot would reduce consumer appetite for pork products and damage the pig industry’s reputation. Concerns are also raised that even with the knowledge there is no cheap and genuinely reliable treatment and individual farms do not directly feel the benefit. If the surveillance data is held privately because of these concerns, it is only useful for understanding swIAV in a particular herd. Collaborative efforts will contribute much more to efforts to control swIAV by the pig farming community.

Surveillance data can be from simple diagnostics such as clinicopathology and post-mortem assessment of a carcass, however, these methods are limited by not providing definitive detection of IAV [86]. For more reliable results, molecular diagnostic tools can assess viral antigens, nucleic acids or host antibodies that bind swIAV. Quantification of swIAV RNA by Reverse Transcriptase quantitative PCR (RT-qPCR) from a nasal swab measures the current viral load. Using conserved primers the RT-qPCR assay can accurately and relatively rapidly detect a broad range of swIAV subtypes [86]. Downstream sequence analysis of amplified viral RNA can also provide insights into the evolution of swIAVs detected and can easily identify genomic reassortment if the full genome is sequenced [51, 92]. The falling costs and increasing ease of nucleic acid sequencing makes it increasingly attractive as a detection method as it has broader benefits of providing insights into the evolution and epidemiology of swIAVs as well as the capacity to identify genomic reassortment events. Current infections can also be detected with non-nucleic acid assays such as an enzyme-linked immunosorbent assay (ELISA) or during post-mortem by immunostaining fixed tissues for swIAV antigens [93].

ELISA can also be utilised to retrospectively assess whether pigs have recently been infected with swIAV [86, 93]. Antibodies circulating in blood plasma can be detected from 1 to 2 weeks post infection and peak after 4–7 weeks [94, 95]. Other retrospective swIAV tests such as the haemagglutination assay and serum neutralisation assays are not available commercially and therefore less relevant for widespread surveillance but have been demonstrated to be effective at distinguishing endemic swIAV strains [94].

A major limitation in testing for serum antibodies to swIAV compared to RT-qPCR is that vaccination stimulates an antibody response and a vaccinated animal cannot be discriminated from a naturally infected animal [86]. Current surveillance measures are also limited because commercially available diagnostic tests are presently only available for specific H1N1 and H3N2 subtypes [86].

### Medical strategies

The application of medical strategies to reduce the prevalence of swIAV is a complementary tool to the traditional strategies and should not be considered a substitute. The epidemiological data from surveillance must be considered to assist the targeted application of medical strategies. Prophylactic treatment for swIAV is performed by individual farms to remove endemic swIAV and thereby improve the productivity of their pigs, however if applied effectively, its broader benefits will contribute to a reduction in the likelihood of transmission between farms, as well as the potential for reassortment and zoonotic transmission [53, 96, 97].

### Vaccination

The principal prophylactic strategy for controlling swIAV is vaccination. As demand for pork products increases, pig production has intensified, and with this the uptake of vaccination to reduce swIAV has as well. After the 2009 Swine ‘Flu outbreak vaccine uptake improved as pig farmers moved to negate the threat of another zoonotic event occurring that had outlined the fragility of consumer confidence in pork consumption and the economic impacts of a swine origin IAV outbreak [16, 98].

The ability of swIAV to evolve by antigenic shift and antigenic drift complicates the creation and vaccine design process, making it essential that effective surveillance is in place to ensure optimal vaccine design. Vaccination has been observed to reduce the reproduction ratio (the number of secondary infections caused by an infected animal) in naïve pigs from 10.66 to below 1, making it an effective strategy for swIAV control [53]. Vaccination for swIAV in swine principally works by inducing the production of virus-specific antibodies via a humoral adaptive immune response which has two mechanisms of function; through antibodies circulating in the host serum that neutralise/opsonise infectious swIAVs and through priming the host immune system to clonally produce antibodies following the detection of the epitope [99]. All licensed vaccines that are currently commercially available are inactivated whole viruses that target the major porcine endemic strains H1N1, H1N2 and H3N2 in various combinations prepared with oil-adjuvants [100]. Because Europe and North America swIAV differ significantly in genetic and antigenic composition, even of the same HA and NA subtype/nomenclature, they must be designed for the prevalent regional strains [101].

The major challenge to effective Influenza vaccination is swIAV’s complex ecology and incessantly mutable genetic and antigenic composition [100]. The more closely matched the vaccine and infectious strain are the better the immune response will be [102] and antibodies will only bind to closely matched target epitopes, thus if a mismatch between the vaccine strains and the infecting swIAV strain is present, serum antibodies will fail in neutralising the infectious virions. HA is by far the most abundant viral protein, comprising about 80% of IAV membrane proteins [103] and is highly accessible to antibody binding as a protruding protein. Because of this, the modus operandi associated with whole inactivated swIAV vaccine mounted responses is to produce antibodies that target the exposed head region of the HA protein [104–106]. Antibody binding with HA interferes with its ability to bind to the host receptor SA, preventing endocytic uptake and therefore viral infection and transmission. However, as the exposed head region of HA is heterogenous (25% genetic distinction within subtypes) and has a high mutation rate, positive selection of swIAV that do not have an HA match with an antibody promotes evolution of swIAV virions that circumvent the vaccine induced immune response [24]. This evolution is a major cause of low vaccine efficacy rates and reason why vaccines must continually be updated and modified to match circulating strains. If antibodies target a more conserved region, such as the stem region of HA they will have activity against a broader range of swIAV and assist in counteracting the circumvention of host immune systems by swIAV evolution [107]. Antibodies have been identified in humans that cross react with the HA stalk region of pH1N1 and also divergent H1N1 and H5N1 influenza strains, suggesting that an effective vaccine could mimic this response in humans at least [108].

Nucleoprotein (NP) and M2 have been considered for vaccine efficacy, however the smaller amount of protein and their localisation make them difficult to effectively target using whole inactivated virus technology [109, 110]. The ultimate goal for Influenza control is for a universal vaccine with reactivity against all strains, however even for humans this is not on the immediate horizon [111].

Using different strains of swIAVs for subsequent vaccination has been observed to protect against both strains better than concurrent bivalent administration [112], however the complexity of distributing specific vaccines strains to different farms/regions for use at specific times makes heterologous vaccination programs administratively difficult and so has led to the less effective but simpler bivalent vaccines becoming ubiquitous. Although swIAV antibodies wane annually and if present will not always match subsequent strains due to HA evolution, there are suggestions that swIAV specific B lymphocytes might be maintained over a pig’s life [99]. In humans, people vaccinated with the 1976 swine-origin H1N1 strain showed a slightly enhanced neutralisation response to the 2009 H1N1 pandemic, albeit in a small cohort [113].

The other major complication for vaccine efficacy is the presence of maternal derived swIAV antibodies in piglets. Typically, 2 doses of vaccine are given intramuscularly to gestating sows prior to farrowing. Although no swIAV specific antibodies transfer through the placenta, maternal antibodies in colostrum confers immunity in neonates and with passive transfer from sows’ milk immunity is usually maintained until around 14–16 weeks old [114, 115]. As neonates do not have a well enough developed immune system to respond well to vaccination this is the most effective way at preventing new-born piglets of becoming a large reservoir for swIAV. As the piglets’ maternal antibody titre declines, susceptible piglets become a reservoir for swIAV, transmitting to other pigs in the herd as their own antibodies wane or the virus evolves [116]. Piglets in comprehensive vaccination programs, are vaccinated at weaning, however the biological variation in reducing antibody titres means that under this system a proportion of piglets will either not have a well enough developed immune system to respond to the vaccine or have no maternal derived antibodies remaining well before vaccination, meaning a naïve reservoir is likely to remain.

The presence of MDAs in piglets presents a conundrum to the farmers. Kitikoon et al. (2006) observed that piglets in their study which had MDAs also had increased infection rates and prolonged presence of clinical symptoms as a result of suppressed serum antibodies and T-cell response compared to piglets without MDAs [104]. An impaired humoral immune response has also been identified after vaccination of piglets against Porcine Reproductive and Respiratory Syndrome Virus (PRRSV) in the presence of MDAs [117].

Furthermore, vaccine-associated enhanced respiratory disease (VAERD) has been experimentally observed whereby more severe disease is caused if the infecting virus does not match the inactived swIAV strains contained in the vaccine due to an obfuscated immune response [118]. As the mechanism for VAERD is not well-understood and it has only been observed under experimental conditions, the consideration it should be given when considering on-farm swIAV vaccination strategy is contested and dependent on the vaccine type [119, 120]. There is a desire to move away from whole inactivated vaccines due to their need for annual updating, inconsistent efficacy in the field and potential VAERD.

An alternative vaccine strategy that is increasingly popular in US commercial settings are autogenous vaccinations, inactivated swIAV isolated from strains endemic to the herd [96, 99]. The major drawback to autogenous vaccines is that they are created from presently circulating strains, and pigs are therefore likely to be immunologically naïve to any new swIAV introductions. However, as commercial vaccines often have low efficacy rates, up to date veterinary knowledge of local swIAV epidemiology can mean autogenous vaccines offer an improved solution to the endemic strains over the broad based commercial vaccines [121]. In a longitudinal study from the USA, there was no significant difference in the reduction of swIAV in herds using either commercial or autogenous vaccinations in sows, however as the autogenous vaccine strains used were not genetically identified, mismatches between the vaccine strain and circulating strains may have reduced the effectiveness of the autogenous vaccination programs [122]. This underlines why it is important to have a robust swIAV surveillance program prior to vaccination to target the right strains and also post vaccination to evaluate if it has been successful and whether the vaccination program needs to be improved.

Vaccination programs in pigs are specific to porcine endemic strains, and therefore will not prevent avian or (most) human IAV strains from infecting pigs. Immunising swine farm employees is important to prevent human to swine transmissions, rather than preventing the zoonotic transmission in to humans [44, 78, 123]. Reducing the potential human-pig transmission diminishes the risk of a swine endemic strain genomically reassorting with the human strain and transmitting back to humans. This was observed in 1998 events when swine viruses acquired human genes, ultimately leading to the emergence of novel swine viruses [80, 87]. Vaccination of pigs against the classical H1N1 swIAV strain did not prevent the emergence of pH1N1, and subsequent transmission between humans and pigs has been identified multiple times [44, 46, 72, 87]. The threat of a swine-origin IAV pandemic emerging from reassorted strains is epitomised with the repeated appearance of Swine ‘Flu outbreaks [124]. With the occurrence of antigenic divergence commercial vaccines need to updated to remain effective against the novel strain. Given that the commercial vaccines are most effective as trivalent concoctions, the addition of another strain would threaten their functionality [99].

Considering that effective vaccination programs require surveillance to be implemented fully effectively, have a cost of labour with two intramuscularly administered doses 3 weeks apart and are likely to entail veterinary consultation, the development of vaccines with broader heterospecific activity and efficacy are essential to improving farmer uptake. Vaccination of piglets at weaning is further complicated by the movement of pigs between breeding and finishing farms, whereby swIAV resistance in piglets does not directly benefit breeding farms as they leave soon after weaning. This results in a dichotomy in the incentives for vaccination between breeders and growers in that the benefit is mostly received by the grower of weaned piglets, but the breeder would ideally vaccinate piglets to reduce the likelihood of swIAV being transported between farms. If the responsibility of vaccination falls on the growing farms, some piglets are likely to already be infected on arrival and will act as a fresh source of swIAV. Because of this there will always be susceptible pigs in the production chain.

For vaccines to be adopted by farmers they need to be cost effective or incentivised in an alternative manner to ensure the broader benefits of controlling IAV ecology are realised. It is notable that in countries that are endemically infected with highly pathogenic avian influenza there are blanket vaccination programs against specific avian IAV strains across complex production systems that could be helpful when considering a framework of how to implement blanket swIAV programs [125, 126].

### Pipeline control measures

The control measures described above work best when applied with coordinated discretion. Likewise, novel strategies for IAV control will operate most effectively when integrated alongside traditional control strategies that are prescribed and implemented in an accurate manner. If infection is prevented, transmission is prevented, hence IAV control is most effective when applied prophylactically. Reactive measures can only be administered when symptomatic signs are seen in an animal or at the herd level, and by that stage of the infective cycle viral shedding has commenced, spreading to naïve hosts who will continue the infective cycle. For a virus that has high rates of subclinical infections the optimal result is to entirely prevent infection, thereby removing a reservoir for the emergence of a human pandemic.

### Genetic technologies

Genetic technologies offer the prospect of broad acting, permanent and heritable resistance to swIAV. Where vaccines are too expensive and there is poor distribution infrastructure the possibility of resistance by selective breeding could be particularly appealing. However, there are no genetic markers currently identified in pigs that could be selected for via traditional breeding programs. Genetic polymorphisms that confer phenotypic resistance to swIAV must therefore be introduced to breeding animals to provide the basis for disseminating resistance by breeding. To reduce the likelihood of mutation escape from genetic resistance in the porcine host, using multiple genetic methods for resistance is recommended to increase the barriers to escape. The discussion of genetic technologies in this review is restricted to relevant viral resistance examples in pigs and how they relate to prospective swIAV resistance.

### Transgenics

Transgenic pigs have been created using various genome engineering technologies to develop in vitro and in vivo porcine models for swIAV resistance. Here, a transgenic animal is defined as one containing DNA not native to that species. Type I Interferon (IFN-I) is an important mediator of the innate immune response to viral infections, and Mx1 (mice and pigs, MxA in humans) is an integral downstream effector protein of the IFN-I antiviral response [127]. Allelic variants of Mx1 confer variable susceptibility to IAV in pigs [128, 129]. Fibroblasts isolated from transgenic pigs generated by Somatic Cell Nuclear Transfer (SCNT) containing multiple copies of porcine Mx1 cDNA were observed to have a 10-fold reduction in IAV titres compared to wildtype controls [130]. IFN-induced Transmembrane Proteins (IFITM) are virus restriction factors stimulated by the innate immune response that inhibit cellular entry of several viral pathogens [131]. Constitutive overexpression of porcine IFITMs in a pig tracheal cell line was observed to reduce IAV infection by Lanz et al. (2015) [132], whilst reduced expression of endogenous IFITMs led to an increase in viral titre. These findings were corroborated Benfield et al. (2015), who reciprocated these findings specifically for IFITM3 [133].

In the fight against PRRSV, Histone deacetylase 6 (HDAC6) has been identified as having anti-viral properties [134, 135]. Lu et al. (2017) [136] created transgenic pigs overexpressing porcine HDAC6 and found PRRSV gene expression was reduced and virus production impeded. These examples of transgenic animals are important in improving our understanding of how we can fight viral infections and the knowledge on these proteins may have applications related to therapeutic drugs. Because constitutively inducing an anti-viral innate immune response is likely to have unintended biological effects on the systemic health of the organism these transgenic strategies are unlikely to ever be realised commercially for ethical reasons.

### RNA interference (RNAi)

A transgenic strategy more plausible in gaining regulatory approval would have specific antiviral activity as opposed to inducing a systemic immune response. Expression of RNA interference (RNAi; short interfering RNA, micro RNAs, short hairpin RNAs) products can be introduced to knockdown transcription or translation of key genes for viral infections [137–139]. In vitro research has observed reductions in gene expression and subsequent impaired replication capacity for swine endemic coronaviruses [140], Classical Swine Fever (CSF) [141], PRRSV [142], African Swine Fever (ASF) [143], Foot and Mouth Disease (FMDV) [144, 145] and IAV of swine and avian origins with RNAi [146]. In vitro success has been translated in vivo, with PPRSV [147], FMDV [148] & CSF [149] transgenic pigs expressing short hairpin RNAs (shRNAs) showing resistance to the relevant viral infection. Importantly, Li et al. (2014) [147] only observed an increase in the IFN-I response when foreign viral RNA is detected and not constitutively against the RNAi molecules. RNAi mediated immunity against IAV has been developed in chickens [150], but has not been demonstrated yet in pigs. However, the success in chickens and the established use of lung specific promoters for RNAi and its effectiveness against other viral pathogens in pigs suggest that it could be a potent inhibitor of swIAV replication with minimal unintended biological consequences. However, the highly mutable nature of swIAV and its genetic heterogeneity would mean transgenic RNAi swIAV resistant pigs would need to target multiple genes to reduce the likelihood of escape by mutation and remain effective, which would increase the risks associated with expressing non-native RNA products by the multitude of transgenic transcripts.

Despite being discussed for use in livestock dating back to at least 2003 [151], it is clear that the promising results of RNAi in research face significant hurdles in transferring the technology to a commercial scenario for public consumption. Delivering the RNAi technology using nanoparticles [152, 153] or viral vectors such as Adeno-Associated Viruses (AAVs) [154, 155] as a therapeutic as opposed to constitutive expression as a defence against swIAV infection could be an alternative delivery mechanism with less regulatory pushback to consider. Transient delivery of RNAi moecules through these methods would permit rapid therapeutic adaptation to the genetic identity of swIAV strains diagnosed in particular regions and would reduce the potential for unintended consequences such as resistance emergence and off-target effects. However, despite the benefits of improved productivity at the farm level and reducing the burden of swIAV in pigs, using RNAi in a therapeutic manner is reactive and would therefore create a reservoir of persistent subclinical infections. For a more comprehensive review of the applications and risks of RNAi in animal agriculture see Bradshaw et al. (2017) [139].

### Gene-editing

A more viable strategy for permanent and heritable resistance than creating transgenic organisms may be in modifying endogenous host genetics to prevent viral exploitation of host proteins. In the microbial evolutionary arms race of bacteria against viruses, we have discovered a molecular mechanism in bacteria that prevents viral infections in a targeted and specific manner. In its native environment, the CRISPR/Cas system functions with a programmable RNA probe that binds to viral nucleic acids through homologous pairing. The RNA probe is complexed with a Cas protein that cuts the target nucleic acid, resulting in degradation of the viral genome. We have extrapolated this microbial technology to create an improved and adaptable gene-editing technology that allows specific changes to be made to mammalian genomes in a time and cost efficient manner [156]. Gene-editing includes deletion of nucleic acids, substitution of nucleic acids or introduction of nucleic acids. Larger scale genome engineering and epigenetic modifications are also possible using the gene-editing toolbox; however, as described here it specifically does not include large non-native regions of DNA being introduced as happens with transgenic animals.

Viruses require a host organism for replication and transmission. This reliance on a host, usually specific in type and/or organism, has led to the evolution of host proteins being exploited during the virus life cycle. By altering the DNA sequence of host genes that code for proteins which are recruited by a virus, such as SA that promotes swIAV endocytosis, we can perturb the viral life cycle, confer resistance to the host and prevent onwards transmission. The crudest form of gene-editing for viral resistance is to delete an entire gene or cause an insertion or deletion (indel) in the coding sequence that introduces a premature stop codon. Introducing an indel into the coding sequence of host cell receptors that lead to phenotypically null pigs has been demonstrated to work effectively for viral resistance to PRRS [157–159] and specific coronavirus strains [160].

A more nuanced approach to prevent PRRSV interaction with the host cell receptor, CD163, was taken by Burkard et al. (2017) [161]. Here, they deleted an exon of the CD163 gene that codes for the protein domain that PRRSV specifically interacts with. Further research of the biological impact of lacking this CD163 domain has thus far identified no unintended biological consequences [162]. The data has not been published for the CD163 null pigs displaying PRRSV resistance and thus unfortunately a complete comparison cannot yet be made between the biological impacts of losing a single exon or the entire protein of CD163. For IAV specific resistance, the host cell receptor (SA) is less appropriate as a target for gene editing due to its crucial role for normal function [163].

A family of nuclear proteins, acidic nuclear phosphatase 32’s (ANP32s) has been identified in chicken and mammalian in vitro studies to have strong pro-viral effects by enhancing the efficiency of IAV genome replication [38]. Human ANP32 null cell lines ablate the infection of swIAVs, and when porcine ANP32s expression is recapitulated with cDNA constructs the ability of the virus to replicate is returned. These findings, corroborated by avian and human in vitro results [164–166], suggest that the conserved gene family of ANP32s supports viral RNA polymerase function and enhance the efficiency of genome replication [167, 168]. Conservation of IAVs exploitation means that successful resistance could be against multiple IAV subtypes to variable extents dependent upon the infected species and IAV polymerase genetics [169]. Specific amino acids in ANP32 proteins that affect the function of viral polymerase activity have been identified to confer a similar reduction in activity as the full loss of function mutations and offer potential targets for substitution [166, 170].

Research and development of the CRISPR/Cas systems has led to a revolution of gene-editing technologies. As a tool for generating research models, Cas9 transgenic pigs and chickens have been developed [171, 172]. Alternative Cas protein-based strategies include using Cas13 which endonuclease activity specifically associated with RNA. A transgenic animal expressing Cas13 and guide RNAs that target the swIAV genome could therefore conceptually be more resistant to infection. Inactivated Cas proteins that have transcription activating or repression domains fused could be therapeutically prescribed to affect the expression of immune response genes [155]. Base-editing and prime-editing are more recent developments that have enhanced the specificity and make small changes to the host genome have not been well assessed in pigs but hold promise for reducing the potential for unintended biological impacts [173, 174].

A major hurdle to the implementation of gene-editing in livestock for disease resistance is gaining regulatory approval and broad public acceptance [175]. From a public health perspective for IAV it is difficult that the application of genome-editing in livestock may only be favoured retrospectively following an outbreak of disease that may have been controllable in the primary instance through genome-editing. Pigs that have no foreign DNA are much more likely to be permitted for animal welfare benefits and economic demands from producers, with regulations in Argentina, Japan and Canada already having legislated to regulate the animal and not the process by which the DNA was altered [176, 177]. Therefore, if the gene-edit has been validated to be benign other than the intended effect they are likely to be allowed for production. These positive steps towards regulatory acceptance alongside the development and optimisation of gene-editing strategies that could be applied at scale in a commercial setting [178] provide optimism for a case by case approach to the acceptance of gene-edited livestock.

Gene-editing could also be applied outside directly editing the infected host genome for vaccine production through changing the genome of chickens that lay eggs used as bioreactors for the propagation of whole IAVs. Ectopic expression of swine factors (such as SA or ANP32s) that support the replication of swine adapted strains in an avian environment could boost the efficiency of replication in eggs, reducing the cost of vaccine production and thereby potentially making uptake of vaccination more accessible. A risk to gene-editing of pigs comes from the corporate nature of pork production, meaning producers that are not integrated with breeding companies offering IAV resistance alleles in their population could lead to IAV risk farms being isolated, causing small holder producers to be perceived as less safe. The economic factions regarding the introduction of new innovative technologies will hopefully not impede improved safety for humans and welfare for pigs from being available.

### Novel immunostimulant strategies

As an alternative to genetic technologies, novel swIAV control strategies being developed are focussed on improving the host immune response through enhanced adjuvants to improve delivery efficacy or alternative vaccine mechanisms that will prime or induce the host immune response [89, 179]. Innovative immune stimulation strategies include novel swIAV vaccine strategies aiming to induce cell-mediated immunity (CMI; T lymphocytes) alongside the antibody mediated response as well as innate immunostimulation therapies. Much of the research into swIAV vaccines piggy backs research into human strategies, in particular research for a universal vaccine that would target all IAV strains. For a full review on novel IAV control strategies in humans see Wei et al. (2020) [180].

Live vaccines are effective, however because they carry a risk of genome reassortment with coinciding infectious swIAV they are not appropriate for commercial use. Richt et al. (2006) [97] demonstrated their effectiveness with a modified live-influenza vaccine based on an H3N2 containing a Non-Structural 1 (NS1) gene expressing a truncated protein. The virus is greatly attenuated in pigs as a result of not being able to suppress the innate immune response without a functional NS1 [181]. Inoculation of pigs with the virus led to complete protection against H3N2 strains, however only partial protection against H1N1 strains, further demonstrating the difficulties faced by swIAV heterogeneity.

The commercially available and autogenous vaccines used in the pig industry are inactivated swIAVs and do not typically stimulate extensive CMI, which is recognised as being more effective against heterologous IAV infections [182]. DNA vaccines circumvent these hurdles as they stimulate both CMI and antibody mediated responses and can be polyvalent in nature by expressing genes of multiple swIAV strains [183–186]. Furthermore, they are comparatively easy to manufacture and DNA can be readily substituted to evolve with swIAV heterogeneity [184]. Experimental results from Karlsson et al. (2018) [187] provides continued promise of realistic DNA doses and intradermal delivery strategies transitioning DNA vaccines into a commercial setting. Administering mRNA that encodes specific antigens of interest has also been demonstrated in pigs to elicit a humoral and cellular immune response [188]. Another method of directing nucleic acids to epithelial cells of the lung is through viral vectors such as recombinant adenoviruses expressing swIAV antigens [189]. It is not known whether these vaccine administration methods will prevent the issue of VAERD but from the smaller samples in research settings it is conspicuously unidentified.

Nucleic acids may not be restricted to being vaccines for therapeutic application. Co-administration of interleukin-6 DNA was observed to enhance anti-IAV activity in mice [183]. It has been observed that SUMOylation changes at the genomic level occur following IAV infection [190, 191]. Schmidt et al. (2020), identified that endogenous retroviral (ERV) expression is enhanced by specific SUMOylation changes, and ERVs that are usually epigenetically silenced are de-repressed with IAV infection following epigenetic reprogramming [192]. Expression of viral dsDNA, albeit endogenous, induces a heightened immune response and is therefore postulated to assist in clearing viral infections. Direct administration of dsDNA to induce the same effect could therefore be a plausible IAV therapy.

Not only are the vaccine stimulants important to improve, but concentrated and timely delivery to lung epithelial cells through improved adjuvants will improve the efficacy of therapeutics. Currently, oil based adjuvants are used with commercial vaccines to improve their immunogenicity [89]. Intranasal administration of immunogenic antigens with a porcine lung surfactant and Poly I:C with inactivated virions have separately been observed as effective in a research setting limited to H1N1 inactivated virions [193, 194]. Intranasal delivery is further supported as a simple and effective administration technique using nanoparticles as a delivery vehicle for M2 antigens [195]. Here, pigs developed immunity to H1N1 through CMI without any detectable antibody response observed. This suggests that combination therapies specifically targeting CMI alongside humoral immunity could be effective.

The adoption and combination of new technologies alongside novel administration methods that reduce the skilled labour and costs required for vaccination will go a long way to improving the uptake from producers and assist in improving control of swIAV.

## Conclusions

swIAV is associated with a considerable burden of disease in pigs that is associated with significant economic effects and represents a major threat to human health. The emergence of the 2009 H1N1 ‘Swine ‘Flu’ pandemic is a warning of the dire impacts that pork production and global food security could incur should a strain emerge again from a porcine host as happened in 1918. With industrial farming practices increasingly adopted and multi-site systems necessitating more pig movement there will be more opportunities for viral reassortment that enhances the potential for novel IAV strains to emerge. Each approach will be variably relevant according to different regions and the dominant farming system, with the available local infrastructure affecting the implementation of each strategy. Implementing effective control measures to reduce the intraspecies and zoonotic spread of swIAV will improve the economic performance of pig production, improve farmer and pig health and negate the potential for pigs to act as a mixing vessel for emergent strains of IAV.

## Data Availability

Not Applicable.

## Abbreviations

IAV: Influenza A virus
IBV: Influenza B virus
ICV: Influenza C virus
IDV: Influenza D virus
swIAV: Swine influenza A virus
PRDC: Porcine respiratory disease complex
VAERD: Vaccine associated enhanced respiratory disease
SA: Sialic acid
ANP32: Acidic nuclear phosphoprotein 32
CRISPR: Clustered regulatory interspaced short palindromic repeats
PB1: Polymerase basic protein 1
PB2: Polymerase basic protein 2
PA: Polymerase
HA: Haemagglutinin
NA: Neuraminidase
NP: Nucleoprotein
M1: Matrix protein 1
M2: Matrix protein 2
NS1: Non-structural protein 1
CMI: Cell-mediated immunity
DNA: Deoxyribonucleic acid
RNA: Ribonucleic acid
HDAC6: Histone deacetylase 6
MDA: Maternal derived antibodies
PRRSV: Porcine reproductive and respiratory virus
SCNT: Somatic cell nuclear transfer
IFITM: Interferon induced transmembrane protein
shRNA: Short hairpin RNA
siRNA: Short interfering RNA
RNAi: RNA interference
pH1N1: Pandemic H1N1
cH1N1: Classic H1N1
GDP: Gross domestic product
TRIG: Triple reassortant internal gene
USDA: United States Department of Agriculture
EU: European Union
CRISPR: Clustered regularly interspaced short palindromic repeats
FMDV: Foot and mouth disease virus
ASF: African swine fever
CSF: Classical swine fever
IFN-I: Interferon-type I
FAO: Food and Agriculture Organisation of the United Nations
OIE: World Organisation for Animal Health
PCR: Polymerase chain reaction
ELISA: Enzyme linked immuno-sorbent assay
AAV: Adeno associated virus
RT-qPCR: Real-time quantitative polymerase chain reaction
